# Management of Symptomatic Horizontal Mid-root Fractures after Unsuccessful Orthograde Endodontic Retreatments

**DOI:** 10.7759/cureus.5473

**Published:** 2019-08-24

**Authors:** Saeed Asgary, Hesam Mirmohammadi, Ardavan Parhizkar

**Affiliations:** 1 Iranian Center for Endodontic Research, Research Institute of Dental Sciences, Shahid Beheshti University of Medical Sciences, Tehran, IRN; 2 Cariology, Endodontology and Pedodontology, Academic Center for Dentistry Amsterdam (ACTA), Amsterdam, NLD; 3 Research Institute of Dental Sciences, Shahid Beheshti University of Medical Sciences, Tehran, IRN

**Keywords:** calcium-enriched mixture cement, cone beam computed tomography, endodontics, horizontal root fracture, periapical periodontitis

## Abstract

This case report describes the management of symptomatic horizontal mid-root fractures in previously traumatized central incisors, which initially had been treated endodontically and retreated afterwards. A 26-year-old female, who had suffered a traumatic injury to the maxillary anterior teeth, was referred owing to the failure of the corresponding orthograde endodontic retreatment and consequent pain/discomfort. Periapical radiographs and cone beam computed tomography showed that both central incisors had horizontal root fractures in their middle third, root canal fillings in their coronal segments, a considerable dislocation of the apical fragments and large radiolucent lesions between their apical segments and coronal fragments. Based on the obtained radiographic and clinical findings, a surgical endodontic retreatment approach for the main segments without removing the apical fragments was applied employing calcium-enriched mixture (CEM) cement as the retrograde biomaterial. Thirty-month clinical and radiographic follow-ups demonstrated successful outcomes. This case report showed the healing potential of horizontal mid-root fractures after surgical endodontics using CEM cement without removing apical fragments.

## Introduction

Horizontal root fractures (HRFs) involve cementum, dentine, dental pulp, bone, and periodontium. HRFs predominantly occur in mature upper incisors [1]. Predisposing factors of HRFs could be related to the person’s anatomic features, i.e., an increased overjet and inadequate lip coverage of upper anterior teeth. When compared with other forms of dental injuries, the incidence of HRFs in permanent dentition is 7.7% of all kinds of dental traumas [1]. HRFs are usually caused by direct physical impact to the tooth, i.e., sport activities, falls, and traffic accidents [2].

Based on the location of the fracture line, HRFs can be classified as cervical, medium, and apical, with possible dislocation of fragments. According to the guidelines presented by the 'International Association of Dental Traumatology (IADT)' for proper diagnosis of HRFs, conventional intraoral radiographs, with several angulations, are routinely recommended. However, cone beam computed tomography (CBCT) can provide enhanced visualization [3].

The prognosis of HRFs is generally acceptable; nevertheless, it depends on the mobility of the coronal segment, location of the fracture line and stage of the root development. Treatment outcomes for HRFs are divided into one of the following categories: i) successful, with three specific radiographic and histological features, and ii) unsuccessful, with interposition of granulation tissue between the fragments [2].

For unsuccessful cases, due to pulp disintegrity/necrosis and bacterial invasion to the area of fracture, root canal therapy (RCT) of the coronal segment should be performed [2-4]. When the coronal segment is dressed with calcium hydroxide, and then followed by obturation with gutta-percha/sealer as the treatment of choice, a success rate of 84% is reported [4].

Failure in the treatment of traumatic injuries may result in further complications. ‘Root Resorption’ (RR) is a frequent complication of traumatic injuries. In majority of cases, pathological RR of dental origin is inflammatory in nature. Root resorption develops when the internal/external protective superficial layer is damaged/altered [5]. When an injury damages the protective layer, i.e. predentine/precementum, inflammation of the pulp/periodontium will induce RR associated with periapical periodontitis [5].

Similar to immature teeth, and owing to loss of the apical stop, next to the short length of coronal fragments, filling/sealing of the coronal segments is often challenging [4]. In 1999, due to its favorable sealing property and biocompatibility, ‘Mineral Trioxide Aggregate (MTA)’ was recommended for the management of necrotic teeth with open apices [6]. MTA has widely changed the traditional approaches as it has shown good clinical results in the treatments of open apices, RRs, root perforations, and inflamed/exposed vital pulps [7, 8]. Currently, MTA is being used in endodontic procedures for the management of intra-alveolar HRFs and orthograde root filling approach [8]. Nevertheless, ProRoot MTA has some drawbacks, i.e., high price, discoloration potential, difficult handling and long setting time [7]. To overcome these problems, several calcium silicate-based materials as bioactive endodontic cements (BECs) have been developed and introduced to the market, e.g. calcium-enriched mixture (CEM) cement, Biodentine, BioAggregate, BioRoot RCS, Endo-CPM, so forth [8].

The aim of this case report was to present the clinical and radiographic findings of previously traumatized and painful maxillary central incisors, which were severely affected by inflammatory root resorption after primary endodontic treatments and subsequent retreatments. Furthermore, this study reported the 30-month prognosis of the performed surgical retreatment, employing CEM cement.

## Case presentation

A healthy 26-year-old female was referred to “Mehr Dental Clinic” for the management of pain/discomfort in her maxillary anterior teeth. Considering the information provided by the patient, the maxillary central incisors had suffered a traumatic injury seven years prior to the initial visit. After a few months, and following the development of an abscess, a general dentist performed RCT for both central incisors. Last year, the patient had again reported pain and symptoms originated from the treated maxillary incisors and thus, she was referred to an endodontist. Then, both incisors underwent an orthograde retreatment with an MTA-like apical plug.

**Figure 1:**
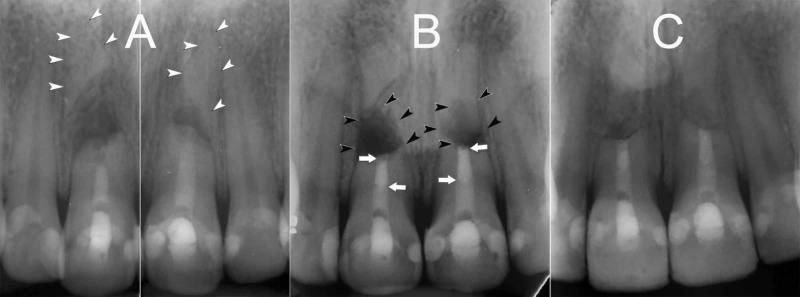
(A) Preoperative periapical radiographs of the fractured maxillary central incisors. (B) Without removing the apical fragments, 3-mm deep root-end cavities on the coronal segments were filled with CEM cement. (C) 30-month recall radiograph; satisfactory healing of the lesions and normal PDLs can be seen (White arrow head = apical segment, Black arrow head = lesion, White arrow = root end filling with CEM). CEM: Calcium-enriched mixture

Clinical examination of central incisors revealed pain to percussion, tenderness/redness on the buccal side of the incisors, normal probing depth, and mobility grade I. Lateral incisors were also examined, and responded positive to sensibility tests. On radiographic examination, the right and left central incisors presented HRFs at their mid-root level with a large dislocation of apical fragments. In addition, short coronal segments with wide-open apices, RRs and inadequate intracanal fillings, with large radiolucent lesions between the coronal and apical fragments, were evident (Figure 1A).

**Figure 2:**
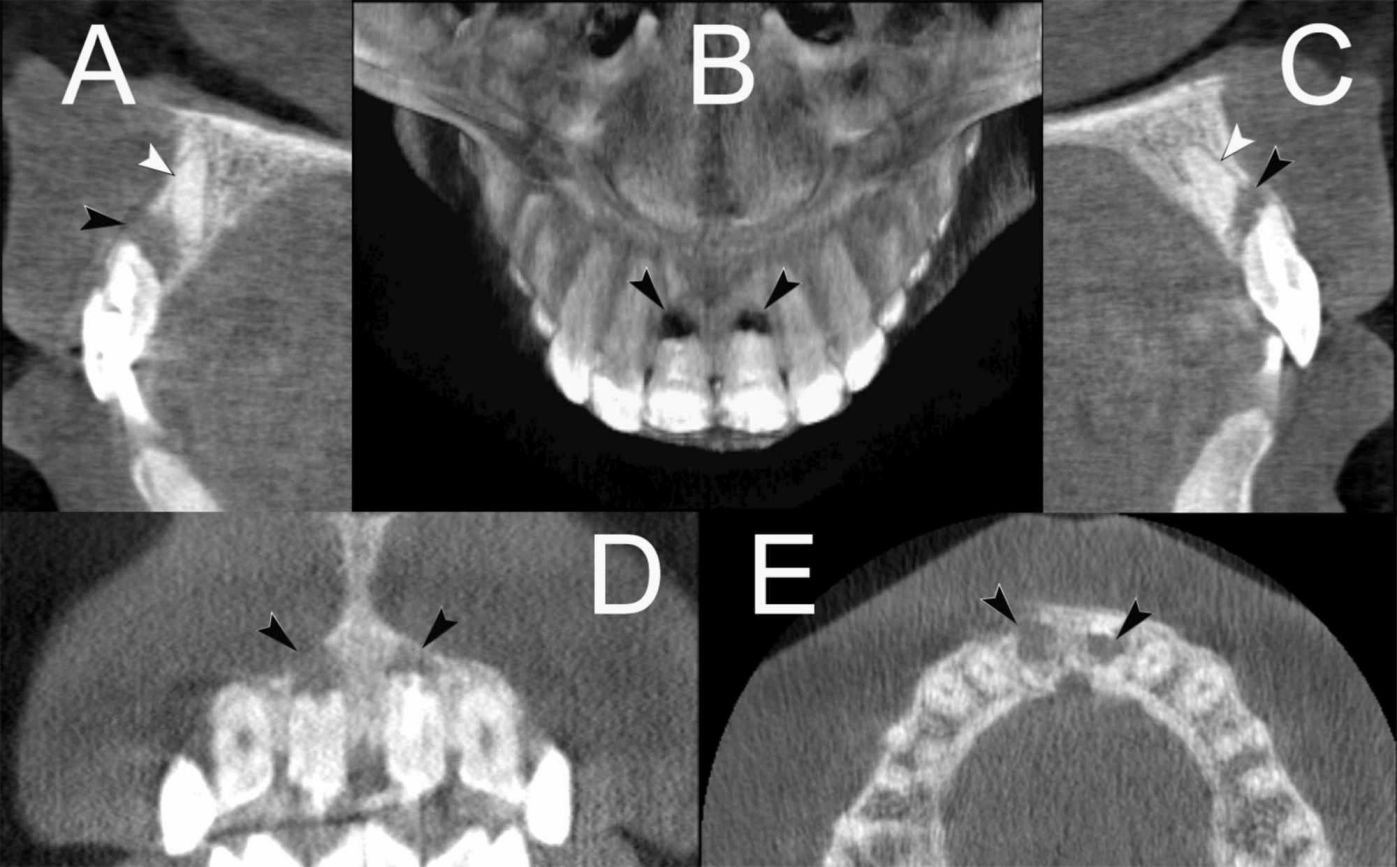
CBCT of the traumatised maxillary central incisors (A & C) through the axial section of the maxillary central incisors revealed oblique root fractures of the middle third, associated with dramatic displacement of the apical fragments. Large radiolucent lesions between the apical and coronal fragments can be seen. Horizontal root fractures at their mid-root level with a large dislocation of apical fragments with large radiolucent lesions between the coronal and apical fragments (A, B, C & E), and short coronal segments with wide-open apices (D) can be seen (White arrow head = apical segment, black arrow head = lesion). CBCT: Cone beam computed tomography

Further analysis with CBCT through the axial section revealed oblique root fractures of the middle third, associated with serious displacement of the fragments in both central incisors. Large radiolucent lesions between the apical and coronal fragments were evident (Figure 2). Clinical symptoms and radiographic signs were consistent with periodontitis, breakdown of bone and external inflammatory RR.

The treatment plan, proposed to the patient, was a) teeth extraction with implant replacement, or b) surgical endodontic retreatment. She chose the latter; therefore, the advantages and disadvantages of the surgery were reviewed, and informed consent was obtained.

Before starting the surgery, the patient was premedicated with a nonsteroidal anti-inflammatory drug (Gelofen, 400 mg), and asked to rinse her mouth with 0.2% chlorhexidine mouthwash. Following the administration of local anesthesia, a sulcular incision, followed by a full rectangular mucoperiosteal flap, was made. Without removing the apical fragments, granulation tissues were removed and sent for further histopathological analysis. With a slight root-end resection, 3-mm deep root-end cavities were prepared using a minipiezon ultrasonic retrotip (Joya electronics, Tehran, Iran) on the coronal segments. CEM cement (BioniqueDent, Tehran, Iran) powder and liquid according to manufacturer’s instructions were mixed and the root-end cavities were filled/sealed with the biomaterial. After radiographic confirmation (Figure 1B), the flap was repositioned, sutured and compressed with a moist gauze for 3 min. She was given verbal and written postsurgical instructions.

Five days later, the patient returned for clinical evaluation and suture removal. The patient was asymptomatic. Histopathological report revealed periradicular granuloma. Thirty-month clinical follow-up showed favorable outcomes. Both central incisors were asymptomatic, functional with no mobility, and had normal probing depths. Radiographically, the lesions were healed, and normal PDLs were established (Figure 1C).

## Discussion

There are various treatment modalities for HRF cases with necrotic pulp. However, surgical endodontics as the last resort is not often considered for the management of unsuccessful endodontically treated/retreated HRFs. In such rare cases, surgical removal of apical fragments, which may compromise the outcomes, is an integral part of the treatment procedure [9, 10]. The main purpose of the mentioned approach is to create an adequate endodontic seal and thus, block the path of communication between the infected root canal system and periradicular tissues at the line of fracture.

In the presented case report, we introduced a surgical intervention for the treatment of HRFs in endodontically treated but failed retreatment, with external RR, and large periradicular lesions that filled the space between the coronal and apical dislocated segments, with no other lesions at the apices of apical fragments.

Throughout the treatment procedure, the involved apical fragments remained intact and root-end fillings were carried out. After a 16-month follow-up, the treated teeth were asymptomatic and functional, and the lesions had healed and been replaced by newly formed bone. It is well-known that the development of a radiolucent lesion, along the line of fracture in HRFs, could be considered as an indicator for pulpal necrosis in the coronal segment, however, and in the apical fragments, the dental pulp usually remains vital [4, 11]. Therefore, almost all researchers recommend RCT only for the coronal segment, not for the apical vital fragments [12]. Consequently, a similar concept in our surgical procedure was followed. Without hard tissue extra-damage, due to the removal of the apical fragments, the healing process improved and favorable clinical outcomes were achieved.

Healing of periradicular periodontitis, as one of the main goals in endodontology following an unsuccessful primary endodontic treatment and orthograde retreatment with creating an apical plug in a non-union HRF, is a challenge. In these circumstances, surgical retreatment, tooth extraction and its replacement with implants are considered the treatment options [11]. In the presented case, saving both maxillary central incisors, due to patient’s esthetic and financial situation, was of great importance. In an instance of single-tooth replacement, the presence of interproximal papillae is predominantly determined by the attachment level of the neighboring teeth, which favors the esthetic outcome of single-tooth replacements in case of periodontally unaffected neighboring teeth. As a result, the presence of a papilla between two implant crowns is predominantly dictated by the highest bone level between the implants, which may jeopardize the esthetic of the patient [13]. A systematic review with meta-analysis showed that in failed RCTs, surgical endodontics was the most cost-effective approach, followed by non-surgical retreatment/crown, tooth extraction/fixed partial denture, and finally tooth extraction/implant-supported restorations [14]. In addition, and from a technical point of view, although the removal of small coronal segments for implant replacement was possibly simple in our case, surgical removal of apical fragments, which needed additional periosteal flap with further bone removal, would have probably been more complex than endodontic surgery. It should be mentioned that, although socioeconomic status of our patient clearly dictated surgical endodontics to save the teeth, implant replacement invariably remained a valuable treatment option.

In the present case, the crown-root ratio could not be recovered to an ideal level, as the root development had already been interrupted, and the inflammatory resorptive process had caused considerable damage to the remaining root structure. Nevertheless, the treatment outcome was considered successful, since the resorption had been arrested and remained stable over the 16-month follow-up period. Furthermore, continued regeneration of the periapical and periodontal tissues was evident and no clinical symptoms were present.

Since the success of an endodontic surgery depends on the selected root-end filling biomaterial, the quality of the chosen material and the consequent hermetic seal are of utmost importance. CEM cement, which is a bioactive endodontic material and mainly consists of CaO, SO3, P2O5 and SiO2, was used as the material of choice in our current case. When CEM cement was applied as a root-end filling, scanning electron microscopy demonstrated a similar distribution pattern of elements in the cement and surrounding dentin [15]. CEM cement releases indigenous calcium and phosphorus ions and is able to precipitate hydroxyapatite over itself, a quality which prepares a good matrix for periradicular tissue regeneration, i.e., periodontal cell attachment, proliferation and cementogenesis [16, 17]. Continuous cementum coverage over CEM cement as a root-end filling material and adjacent dentin is an important eminence, and thus, it can serve as a barrier against the destructive residual content within the root canal system [17]. In addition, histological studies have revealed that after root-end filling or repair of perforations with CEM, cementum deposition occurred over the biomaterial [7, 17]. Furthermore, clinical trials have shown ≥90% success rate for periradicular surgery or intentional replantation using CEM cement [18, 19].

## Conclusions

In conclusion, traumatised teeth with mid-root fractures have shown healing potential after an initial treatment failure. In such cases, endodontic surgical retreatment, without the removal of the apical fragment, can be considered as a successful approach. CEM cement as a root-end filling biomaterial has exhibited favourable results.
